# The Chin as an Index of Character

**Published:** 1897-12

**Authors:** 


Ernest Crutchen, M. D., of Great Falls, Montana, publishes the following in the *Medical Council*, on "The chin as an index of character:"

*To the Medical Council:*

I have just read Dr. Leuf's letter on "Physiognomy" in the *Medical Council*, and am prompted to say in reply to his request for contributions on the "Chin:"

Protruding chins characterize men and women of the get-there type. Successful people usually carry their chins thrust forward, with compressed lips. This chin, if heavy, with broad rami and swelling masseters, indicates fighting blood.

A retreating chin shows lack of force, mentally, morally, and physically. Usually of the sweet, yielding sort; soon discouraged; desire protection; small executive force. The development of other faculties often makes up for this defect.

A small, well-rounded chin, with a mobile and red cushion of flesh upon it, indicates a pleasure-loving owner. If dimpled, all the more so, for dimpled chins belong to coquettes. People with dimples love to be petted and loved; like admiration and praise. Generally fickle. Usually this chin is healthy, recuperative and long-lived.

Broad chins signify nobleness and large dignity, unless vertically thin, when, if with it there be thin lips of bloodless kind, you find cruelty.

Square chins with little flesh denote firmness and executive ability. These make good haters.

Drunkards usually have a circular line about their chins.

Slovens have wrinkles about their chins.

Long thin chins are poetical, unstable and delicate in constitution. Such people are subject to bowel derangements. If thin through the angles of the mouth, too, they are prone to tuberculosis. Generally short-lived

Medium chins with a suggestive bifurcation in the centre, with small mounds of flesh on either side, characterize generosity, impulsiveness, cheery natures. (The same sized chins, with a dab of flesh just under the centre of lower lip, indicate meanness, selfishness, brutality.)

N. B.—No one feature can be taken in judging character. Often development of other faculties of mind or feature entirely govern. In each case take the "totality of indications" before judging.

Yours very truly,

Great Falls, Montana. ERNEST CRUTCHEN M. D.

[ It does seem as if we should have no trouble to read one's character. Compare your wife's or sweetheart's chin with the above and then, if not satisfied, we refer you to the numerous articles written on "how to read characters and dispositions through the medium of the eye, nose, mouth, ear, head, feet, and in fact any old part of the anatomy, which will give you just the information you are looking for."—ED.]

